# Comparative evaluation of the therapeutic effect of metformin monotherapy with metformin and acupuncture combined therapy on weight loss and insulin sensitivity in diabetic patients

**DOI:** 10.1038/nutd.2016.16

**Published:** 2016-05-02

**Authors:** A Firouzjaei, G-C Li, N Wang, W-X Liu, B-M Zhu

**Affiliations:** 1 Key Laboratory of Acupuncture and Medicine Research of Minister of Education, The Second Clinical School, Nanjing University of Chinese Medicine, Nanjing, China; 2 Epidemiology and Biostatistics Department, Nanjing University of Chinese Medicine, Nanjing, China

## Abstract

**Objective:**

Obesity induces insulin resistance (IR), the key etiologic defect of type 2 diabetes mellitus (T2DM). Therefore, an incidence of obesity-induced diabetes is expected to decrease if obesity is controlled. Although Metformin is currently one of the main treatment options for T2DM in obese patients, resulting in an average of 5% weight loss, adequate weight control in all patients cannot be achieved with Metformin alone. Thus, additional therapies with a weight loss effect, such as acupuncture, may improve the effectiveness of Metformin.

**Subjective:**

We designed this randomized clinical trial (RCT) to compare the effects of Metformin monotherapy with that of Metformin and acupuncture combined therapy on weight loss and insulin sensitivity among overweight/obese T2DM patients, to understand whether acupuncture plus Metformin is a better approach than Metformin only on treating diabetes. To understand whether acupuncture can be an insulin sensitizer and, if so, its therapeutic mechanism.

**Results:**

Our results show that Metformin and acupuncture combined therapy significantly improves body weight, body mass index (BMI), fasting blood sugar (FBS), fasting insulin (FINS), homeostasis model assessment (HOMA) index, interleukin-6 (IL-6), tumor necrosis factor-α (TNF-α), leptin, adiponectin, glucagon-like peptide-1 (GLP-1), resistin, serotonin, free fatty acids (FFAs), triglyceride (TG), low-density lipoprotein cholesterol (LDLc), high-density lipoprotein cholesterol (HDLc) and ceramides.

**Conclusions:**

Consequently, Metformin and acupuncture combined therapy is more effective than Metformin only, proving that acupuncture is an insulin sensitizer and is able to improve insulin sensitivity possibly by reducing body weight and inflammation, while improving lipid metabolism and adipokines. As a result, electro-acupuncture (EA) might be useful in controlling the ongoing epidemics in obesity and T2DM.

## Introduction

Type 2 diabetes mellitus (T2DM) is a metabolic disorder of fuel homoeostasis caused by islet β-cells. Diabetes develops because of inadequate islet β-cell and adipose-tissue responses to chronic fuel excess, which results in so-called nutrient spillover, insulin resistance (IR) and metabolic stress.^1^ The incidence of T2DM has risen dramatically to an estimated 30 million adults in 1985 and its grew to 135 million in 1995 and reached 173 million in 2002. It is predicted T2DM reach in 366 million adults in 2030.^2^ In fact, the obesity epidemic appears to be driving the T2DM epidemic in parallel. Obesity is a key risk factor for T2DM as it lowers insulin sensitivity in peripheral tissues and induced IR^3^—just elevating body mass index (BMI) from 27 to 29.9 can result in serious metabolic complications.^4^ IR is a multi-factorial condition in which overnutrition triggers increased inflammation and impaired lipid metabolism, characteristic features of T2DM.

No new insulin sensitizer has been invented since 1997. However, Metformin monotherapy, a first-line therapy for T2DM, has not shown strong effects on obesity and T2DM, and the combination of Metformin with other therapies may be more effective. Meanwhile, conventional approaches to weight loss, such as diet and physical exercises, still cannot achieve adequate weight control in all T2DM patients. Thus, we suggest that acupuncture, as a complementary therapy, can be used to obtain better results and aid in improving the efficacy of Metformin. Acupuncture is one of the oldest healing practices and is recognized by the National Institute of Health and the World Health Organization.^5, 6^ According to the theory of traditional Chinese medicine, acupuncture regulates 'Qi and Blood' and is likely to affect the bioavailability of substances taken internally and, in the process, may influence the absorption, distribution, metabolism and/or excretion of substances.^7^ The effect of acupuncture on T2DM, obesity, plasma free fatty acids (FFAs) level and on insulin sensitivity has been addressed in experimental and clinical studies.^8, 9, 10, 11, 12, 13^

There are several parameters that indicate the relationship between obesity, IR and T2DM. Obesity causes a low-grade inflammation and is associated with a chronic inflammatory response and increased levels of tumor necrosis factor-α (TNF-α), interleukin-6 (IL-6) and/or C-reactive protein (CRP). Obesity then alters insulin sensitivity by triggering different key steps along the insulin-signaling pathway. For example, obesity causes IL-6 to have a compensatory role, increasing islet glucagon-like peptide-1 (GLP-1) production, which augments insulin secretion in order to adapt to IR and prevent T2DM.^14^ In addition, obesity can alter adipokine production, which regulates body weight, appetite and energy expenditure and modulates IR. For example, leptin, which controls TNF-α production,^15^ has been implicated in the modulation of glucose homeostasis and IR;^16^ adiponectin, which is the most abundantly secreted protein by white adipose tissue, has a well-known inverse relationship with adiposity and risk of T2DM. Adiponectin is also inversely associated with IR in human populations and some studies have demonstrated that adiponectin exerts an insulin-sensitizing effect;^17^ elevated resistin has been shown in obese rodents.^15^ In the other hand, increased levels of circulating FFAs and dyslipidemia can be found in T2DM patients; and increased ceramides biosynthesis induced by TNF-α or saturated FFAs seems to impair insulin action.^18^

We designed this randomized clinical trial (RCT) to compare the therapeutic effects of Metformin monotherapy with Metformin and acupuncture combined therapy on weight loss and insulin sensitivity. We compared the inflammatory markers, lipid profiles and adipokines in overweight/obese T2DM patients receiving the combined therapy with those receiving the Metformin monotherapy, to understand whether acupuncture plus Metformin is a better approach than Metformin only on treating diabetes.

## Materials and methods

### Ethic review and approval

This trial is registered in ClinicalTrials.gov, NCT02438540. It follows the principles of the Declaration of Helsinki (Version Edinburgh 2000). It has been reviewed and approved by the Ethics Committee Board of Pardis multiple pains clinic (Approved No. 20140713). Before randomization, all participants were requested to sign a written informed consent to decide whether they were willing to participate in this trial. They were informed of the details of the study and all benefits and risks of participating in this trial.

### Study design and population

**Figure 1:**
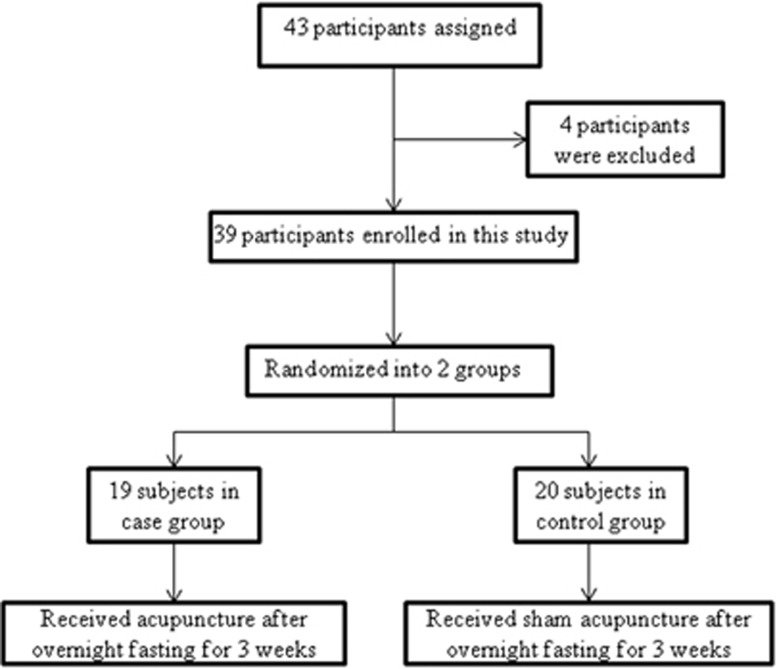
Trial profile and design.

Sample size is calculated based on some RCTs, as a reference in this field, where their sample size was 31,^7^ and according to this statistic basic formula:^19^


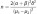


where *n* is the sample size of case group/control group, *α* is the significance level, *β* is the power of test, *δ* is the population variance (s.d.) and *μ*_1_−*μ*_2_ is the smallest effect of interest. In this study, *α*=0.05, *β*=0.10 (90%), *δ* is estimated at 0.40 and *μ*_1_−*μ*_2_ for homeostasis model assessment (HOMA) index (the most important index to evaluate insulin sensitivity) is estimated at 0.60 (minimum clinical significance value for HOMA index, which can be confirmed by clinical doctor, similar to some RCT's^20^), based on the primary clinical data; therefore, *n*≈10 is in each group. A total of 43 participants initially took part in our randomized double-blind (subject and assessor) placebo-controlled clinical trial. Four of them were excluded from the trial according to the inclusion and exclusion criteria. As a result, 39 participants were distributed by permuted-block randomization. In all, 19 participants were assigned to the case group and 20 to the control group by an independent research assistant, and according to the above-mentioned method of randomization. All patients were blinded to the treatment assignments during the period of study and the assessor was blinded to patients' information, including their names and the results from the measurements (Figure 1).

### Inclusion criteria

Participants were included in this study if they fulfilled all of the following conditions: (1) Patients between 20 and 65 years old of either gender; (2) Patients diagnosed with T2DM and had been using Metformin monotherapy as well to control their diabetes during the period of this study as previously (500 mg one/two/three times per day). (3) Patient assessments were compatible with the diagnostic criteria for T2DM set by the American Diabetes Association (Symptoms of diabetes plus random blood glucose concentration ⩾11.1 mmol l^−1^; fasting plasma glucose ⩾7.0 mmol l^−1^; 2-h plasma glucose ⩾11.1 mmol l^−1^, during an oral glucose tolerance test). (4) Patients were overweight with a BMI of ⩾25.

### Exclusion criteria

Patients with any of the following conditions will be excluded from the study: (1) Individuals with nephrotic syndrome (urine protein over 3.5 g day^−1^), edema or renal failure (serum creatinine over 115 μmol l^−1^). (2) Individuals diagnosed with heart failure (NYHA Fc III-IV) or with an implanted pacemaker. (3) Individuals with liver dysfunction (aspartate amino-transferase and glutamate pyruvate transaminase levels twofold above the normal range) or a diagnosis of cirrhosis. (4) Individuals with a high hemoglobin A1C level (hemoglobin A1C above 9%). (5) Pregnant or nursing mothers, including those who have given birth in the last 6 months. (6) Individuals already receiving insulin therapy. (7) Individuals receiving other therapy or had any change in dosage during the period of therapy. (8) Individuals suffering from endocrine abnormalities such as thyroid disease and polycystic ovarian syndrome. (9) Individuals receiving weight loss supplements, anti-depressant agents or hormonal medicine during the last 3 months and the period of the study. (10) Individuals suffering from homeostasis disorders or other systemic diseases. (11) Individuals who did not comply (signed informed consent) with the treatment during the study period.

### Methods

For electro-acupuncture (EA), we used normal EA machine (Hwato brand, SDZ-II, Suzhu, Jiangsu, PR China), electric lines connected to eight needles, with a frequency of 15 Hz/10 mA, at points: Tianshu (ST 25), Daheng (SP 15) and Shuidao (ST 28), bilaterally. Red or positive poles were located on the right side, and at zhongwen (REN 12); black or negative poles were located on the left side, and at Qihai (REN 6). EA was applied for 30 min. Copper acupuncture needles (0.25 mm × 0.40 mm/32 Gauge, EACU brand, Maanshan, Anhui, China) were used in this trial.

### Acupoints

Ten body acupoints were chosen in this trial for both groups. (1) Zhongwan (REN 12) on the anterior midline, 4 cun above the umbilicus; (2) Tianshu (ST 25), 2 cun lateral to the center of the umbilicus; (3) Zusanli (ST 36), one finger-width (middle finger) from the anterior border of the tibia; (4) Sanyinjiao (SP 6), 3 cun directly above the tip of the medial malleolus, and posterior to the medial border of the tibia; (5) Shuifen (REN 9), on the anterior midline, one cun above the umbilicus; (6) Hegu (LI 4), on the dorsum of the hand, between the first and second metacarpal bones; (7) Daheng (SP 15), 4 cun lateral to the corner of umbilicus; (8) Shuidao (ST 28), 3 cun below umbilicus, and 2 cun lateral to Guanyuan (REN 4); (9) Quchi (LI 11), on the flexed elbow in the depression point at the lateral end of the transverse cubital crease; (10) Qihai (REN 6), on the anterior midline, 1.5 cun below the umbilicus.

Ear acupoints chosen were Sanjiao, Jidian (Hunger), Wei (Stomach), Shenmen, Neifenmi (Endocrine) and Pi (Spleen).

### Materials

All participants were treated 10 times every other day, for a total duration of 3 weeks. In the case group, enrolled participants were treated with Metformin monotherapy and received EA, and Auricular acupuncture (Aa), at the selected acupoints. Needles were inserted into the muscle layer at the selected points, and no specific manipulation was carried out; they were only inserted into a specific depth, at which the patients experienced a sensation of de-qi, and electrical stimulation was applied. In the control group, participants were treated with Metformin monotherapy and received sham EA and sham Aa treatment. For those points that were located in the abdomen, needles were inserted 0.3 cm laterally from the acupoints, and the needling was maximally superficial. For the points that were located on other parts of the body, needles were inserted 0.5 cm up and 0.5cm laterally from the acupoints, and needling was superficial as well. In this group, electric lines were connected to some of the needles in the same way they were in the case group. The EA machine was switched off during the 30-min therapeutic time. For Aa, we just used sticky layers without seeds.

### Clinical evaluation

IR was calculated by the homeostasis model (HOMA-IR), proposed by Matthews *et al.* HOMA-IR=(fasting insulin (mmol l^−1^) × fasting glucose (μIU ml^−1^))/22.5; body height was measured to an accuracy of ±0.1 cm (body weight was measured while the subjects were dressed in light clothing after an overnight fasting and by a standard scale to an accuracy of ±0.1 kg); BMI was calculated by dividing weight (kg) into height (squared m^2^). Blood markers including fasting blood sugar (FBS), fasting insulin (FINS), IL-6, TNF-α, CRP, leptin, adiponectin, resistin, GLP-1, HOMA index, FFAs, low-density lipoprotein cholesterol (LDLc), high-density lipoprotein cholesterol (HDLc) and ceramides were calculated by measuring them after drawing 10 ml of blood from cubital vein in each patient, after 8 h of overnight fasting and before treatment; blood was collected three times during the study (at the beginning, at the fifth time and at the end), from groups, using standard range and ELISA diagnostic kits (Biomatik USA LLC, www.biomatik.com), and clinical assessments and measurements undertaken after 3 weeks. Readings/assessment was performed at 3 weeks.

### Statistical analysis

All data were managed by Epidata software, version 3.1 (The EpiData Association, Odense, Denmark, 2003-2005), then analyzed by SPSS software 15.0.0 (SPSS Inc., Chicago, IL, USA) (6 September 2006). Our statistical methods included *T*-test, and repeated measures ANOVA. *P*-value <0.05 was considered as statistically significant.

## Results

The average ages of our 39 participants were 42.2 in the case group and 40.5 in the control group. Eight members of the case group were male and eleven were female. In the control group, 8 members were male and 12 were female. No statistical difference in sex or age was observed among these case and control groups. Clinical and biochemical characteristics of participants have been summarized in Table 1.

### Effect of combined therapy on body weight and BMI

**Figure 2:**
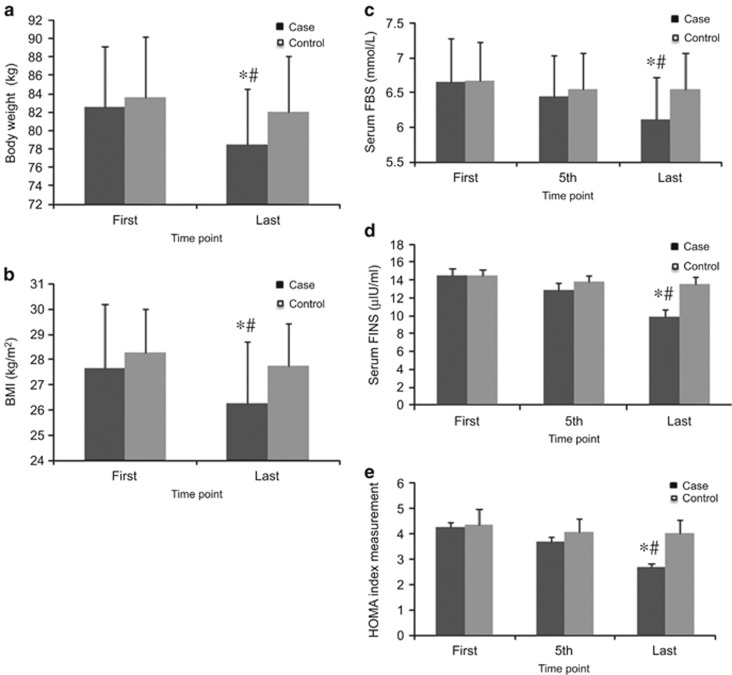
Effects of EA treatment on anthropometric and T2DM markers. (**a**) Body weight (±0.1 kg). (**b**) BMI (kg m^−^^2^). (**c**) Serum FBS concentration (mmol l^−1^) by ELISA. (**d**) FINS concentration (μIU ml^−1^) by ELISA. (**e**) HOMA index=FBS × FINS/22.5. Data were expressed as means±s.d., *P<0.01 vs Control, ^#^*P*<0.01 vs First. Time point: First: at the beginning of treatment, 5th: at the fifth time, last: at the last time of treatment.

Body weight and BMI reductions were observed in both groups. However, significant differences between the changes in body weight and BMI (*P*<0.001) were found only in the case group. The changes in body weight (82.6±6 kg before treatment to 78.4±6 kg after treatment, *P*<0.001) and BMI (27.6±2.5 before treatment to 26.2±2.4 after treatment, *P*<0.001) were significant only in the case group, compared with the control group (Figure 2).

### Effect of combined therapy on T2DM markers

The FBS marker had changed in both groups, but significant glucose-lowering activity was noted only in the case group (from 6.65±0.6 mmol l^−1^ before treatment to 6.12±0.5 mmol l^−1^ after treatment, *P*<0.001). In addition, there was a significant difference in the glucose-lowering activity between the case and control groups (*P*<0.001). As for FINS, significant insulin plasma levels were observed only in the case group (from 14.47±0.8 μIU ml^−1^ before treatment to 9.91±0.7 μIU ml^−1^ after treatment, *P*<0.001). There was a remarkable difference in the FINS level between the case and control groups (*P*<0.001). Simultaneously, HOMA index was significantly changed only in the case group (from 4.25±0.2 before treatment to 2.67±0.1 after treatment, *P*<0.001), and there were significant changes between the two groups in HOMA index (*P*<0.001) (Figure 2).

### Effect of combined therapy on Lipid profiles

**Figure 3:**
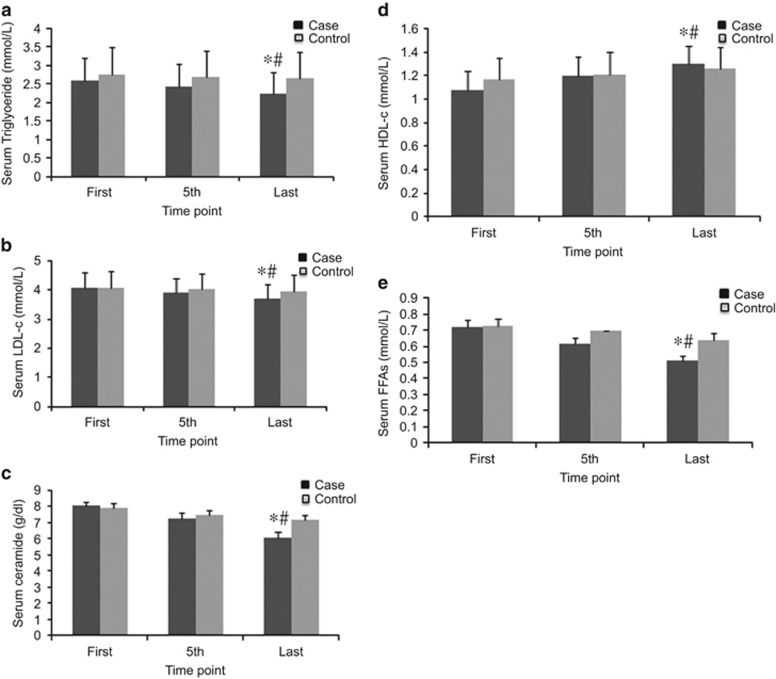
Effects of EA treatment on lipid profiles. (**a**) Serum TG (mmol l^−1^). (**b**) Serum LDLc (mmol l^−1^). (**c**) Serum ceramide (g dl^−1^). (**d**) Serum HDLc (mmol l^−1^). (**e**) Serum FFAs (mmol l^−1^). All data by ELISA. Data were expressed as means±s.d., *P<0.01 vs Control, ^#^*P*<0.01 vs First. Time point: First: at the beginning of treatment, 5th: at the fifth time, last: at the last time of treatment.

FFA levels were reduced in both groups; however, there was a significant difference in the reduction of FFA levels between the case and control groups (*P*<0.001). The reduction was significant only in the case group (from 0.72±0.4 mmol l^−1^ before treatment to 0.51±0.3 mmol l^−1^ after treatment, *P*<0.001). Remarkable difference in plasma triglyceride (TG) lowering activity was noted between the two groups (*P*<0.001) as well. TG levels were reduced in both groups; however, the reduction was significant only in the case group (from 2.59±0.6 mmol l^−1^ before treatment to 2.24±0.6 mmol l^−1^ after treatment, *P*<0.001). Reduction of LDLc levels was also observed in both groups, but was significant only in the case group (from 4.05±0.5 mmol l^−1^ before treatment to 3.68±0.5 mmol l^−1^ after treatment, *P*<0.001). In addition, HDLc levels were elevated in both groups, while this change was markedly different between the case and control groups (*P*<0.001), and was significant only in the case group (1.08±0.1 mmol l^−1^ before treatment to 1.29±0.1 mmol l^−1^ after treatment, *P*<0.001). Finally, ceramide levels were reduced remarkably only in the case group (8.06±0.2 g dl^−1^ before treatment to 6.06±0.3 g dl^−1^ after treatment, *P*<0.001), and significant difference in lowering ceramides plasma activity was observed between the case and control groups (*P*<0.001) (Figure 3).

### Effect of combined therapy on inflammatory markers

**Figure 4:**
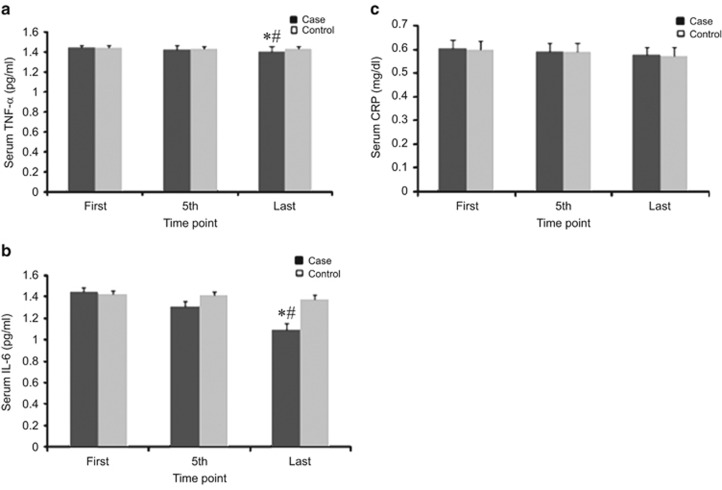
Effects of EA treatment on inflammatory markers. (**a**) Serum TNF-α (pg ml^−1^). (**b**) Serum IL-6 (pg ml^−1^). (**c**) Serum CRP (mg dl^−1^). All data by ELISA. Data were expressed as means±s.d., *P<0.01 vs Control, ^#^*P*<0.01 vs First. Time point: First: at the beginning of treatment, 5th: at the fifth time, last: at the last time of treatment.

TNF-α levels were reduced in both groups, but a significant reduction was noted only in the case group (from 1.44±0.2 pg ml^−1^ before treatment to 1.41±0.5 pg ml^−1^ after treatment, *P*<0.001). In addition, there was a significant difference in anti-inflammatory activity (TNF-α reduction) between the case and control groups (*P*=0.04). IL-6 levels were reduced in both groups, but a significant reduction was noted only in the case group (from 1.44±0.3 pg ml^−1^ before treatment to 1.09±0.5 pg ml^−1^ after treatment, *P*<0.001). In addition, there was a significant difference in anti-inflammatory activity (IL-6 reduction) between the case and control groups (*P*<0.001). Interestingly, CRP levels were not significantly reduced in either group (in the case group: from 0.60±0.03 mg dl^−1^ before treatment to 0.57±0.03 mg dl^−1^ after treatment, *P*>0.05; in the control group: from 0.59±0.03 mg dl^−1^ before treatment to 0.57±0.03 mg dl^−1^ after treatment, *P*>0.05). Thus, there was no significant difference in CRP levels between the two groups (*P*=0.882) (Figure 4).

### Effect of combined therapy on adipokines

**Figure 5:**
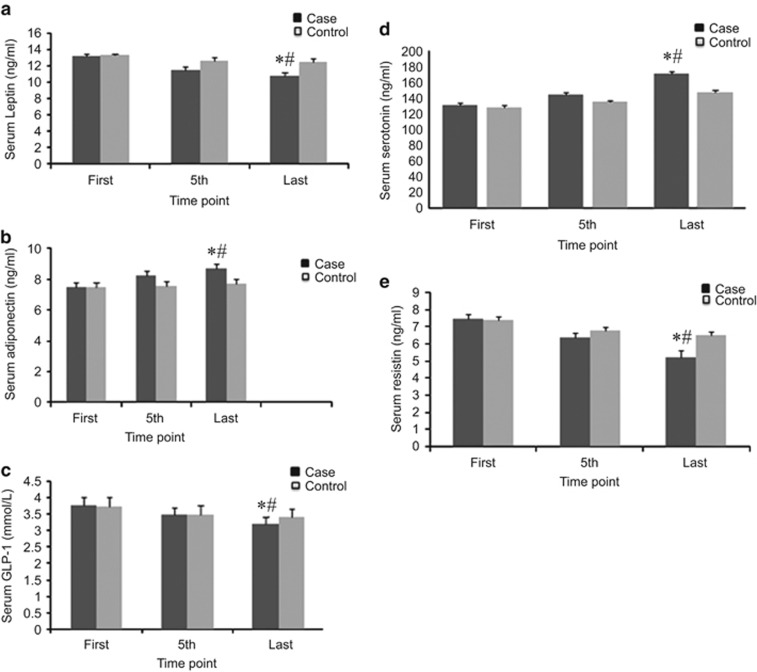
Effects of EA treatment on adipokines. (**a**) Serum leptin (ng ml^−1^). (**b**) Serum adiponectin (ng ml^−1^). (**c**) Serum GLP-1 (mmol l^−1^). (**d**) Serum serotonin (ng ml^−1^). (**e**) Serum resistin (ng ml^−1^). All data by ELISA. Data were expressed as means±s.d., *P<0.01 vs Control, ^#^*P*<0.01 vs First. Time point: First: at the beginning of treatment, 5th: at the fifth time, last: at the last time of treatment.

There was a significant difference in changes, in leptin levels between the two groups (*P*<0.001), and leptin levels were significantly reduced only in the case group (from 13.32±0.2 ng ml^−1^ before treatment to 10.84±0.4 ng ml^−1^ after treatment, *P*<0.001). A significant difference was noted between adiponectin levels in the case and control groups (*P*<0.001); adiponectin levels were elevated remarkably only in the case group (7.47±0.2 μg ml^−1^ before treatment to 8.73±0.2 μg ml^−1^ after treatment, *P*<0.001). GLP-1 levels were also significantly reduced only in the case group (3.77±0.2 mmol l^−1^ before treatment to 3.21±0.1 mmol l^−1^ after treatment, *P*<0.001); there was a significant difference in the GLP-1 levels reduction between the two groups (*P*<0.001). In addition, resistin levels were significantly reduced only in the case group (from 7.43±0.2 ng ml^−1^ before treatment to 5.24±0.3 ng ml^−1^ after treatment, *P*<0.001), and there was a significant difference between the case and control groups (*P*<0.001). Finally, serotonin levels were significantly increased only in the case group (from 130±3 ng ml^−1^ before treatment to 170±3 ng ml^−1^ after treatment, *P*<0.001) and there was a significant difference between two groups (*P*<0.001) (Figure 5).

## Discussion

The results of this study show improvement in all measured parameters in both the case and control groups, but the changes were significant only in the case group, in which participants were treated with true acupuncture. However, the changes in CRP level were not significant. The effects of the combined therapy on the measured parameters have been summarized in Figures 2, 3, 4, 5.

Our results indicate the greater efficacy that EA has in the reduction of body weight and BMI in the case group compared with that of the control group. A systematic review of the 44 trials of acupuncture treatment has concluded that acupuncture is more effective than placebo or lifestyle modification in reducing body weight, and is as efficacious as conventional anti-obesity drugs.^5^ On the other hand, some studies have reported no significant effect in the acupuncture treatment of obesity; however, it should be noted that these studies used Aa, as the treatment method.^21, 22, 23^

The changes in body weight and BMI in this study prove that acupuncture-combined therapy is efficient in achieving weight loss. However, the mechanism of EA in weight loss remains in question. It is thought that acupuncture exerts its effects on weight loss through different mechanisms, such as reducing hunger, affecting lipid and carbohydrate metabolism, affecting activity of glucose-inhibited neurons and modulating feeding behavior.^24, 25^

To understand the weight loss mechanism of acupuncture, we evaluated some important parameters that have been used for studying obesity. As obesity is a low-grade inflammation, and EA has shown to have an anti-obesity effect,^26^ we measured important inflammatory markers such as TNF-α, IL-6 and CRP, in our patients, during EA treatment. Positive changes in measured inflammatory markers in both case and control groups were observed, but these changes were significant only in the case group, in which the patients were treated by true acupuncture. Although there is a correlation in healthy individuals between BMI and CRP levels, CRP is known to be an independent parameter.^15^ Abdi *et al.*^26^ assessed the role of acupuncture on CRP levels during weight loss, in which CRP did not significantly change between two groups; our findings were consistent with these data and did not show changes in CPR levels. In another study, EA plus diet adjustments decreased the levels of serum TNF-α^27^ our study showed similar results. In addition, adipocytes are known to contain higher level of IL-6 in obese rodents; Manneras *et al.*^17^ assessed the effect of EA in adipose tissue in rats with poly cystic ovarian syndrome, in which, IL-6 expression tended to be restored after EA. Meanwhile, EA reduced serum IL-6 concentration, similar to our results. Therefore, we conclude that acupuncture treats obesity at least partially, by enhancing the anti-inflammatory mechanism.

Given that pro-inflammatory cytokines are implicated in the pathogenesis of IR,^28^ increased levels of TNF-α and IL-6 in obese patients can alter insulin sensitivity by triggering different key steps in the insulin-signaling pathway.^29^ Recent studies have suggested that IL-6 could be involved in IR and the downregulation of GLUT4.^13^ In our study, we sought to understand the relationship between weight loss, inflammatory markers and IR, when T2DM patients were treated with acupuncture-combined therapy. A study by Liao *et al.*^17^ has reported a glucose-lowering effect of EA+metformin through activation of GLUT4 and upregulation of MAPK expression in insulin-resistant rats. They concluded that EA+metformin facilitated insulin sensitivity. In similar, our results support our hypothesis in that acupuncture and Metformin combined therapy is more effective than Metformin monotherapy, in inhibiting inflammation, which in turn may improve insulin sensitivity. Consequently, acupuncture's effects on weight loss may be explained through its anti-inflammatory mechanisms.

We also measured other parameters that can be influenced by obesity, such as FBS, FINS and HOMA. Our results show that acupuncture and Metformin combined therapy is able to significantly reduce levels of FINS. For example, β-cells compensate for IR by increasing insulin secretion, and as insulin increases, IR becomes more dramatic. Under these circumstances, EA may be able to provide relief to the pancreas stress that may lead to pancreas failure. Moreover, this combined therapy has the potential benefit of extending the time before pancreatic β-cell failure occurs with T2DM patients, rather than of decreasing IR. FBS levels were also reduced in both case and control groups; however, this reduction was significant only in the case group, suggesting that EA may be able to produce a hypoglycemic response. As a result, EA possibly acts as a kind of insulin sensitizer. Our findings are similar to Cabioglu's findings^30^ that show a decrease in blood glucose levels in obese women, who are treated by EA in combination with a calorically restricted diet. However, a study by Szczudlik *et al.*^31^ has shown no difference in blood glucose levels in patients, who had received EA; however, using EA for only a short time (15 min), in different acupoints, and for a small population (10 patients), may explain the ineffectiveness. In addition, HOMA index, which is belonging to FBS and FINS indexes, remarkably decreased in the case group, accordingly. Meanwhile, IR was reversed by EA and Metformin combined therapy. This result is compatible with Lin's report ^7^ that shows that insulin sensitivity is enhanced by EA and TZD combined therapy, in comparison with TZD monotherapy, when using the HOMA index as the marker. Another study by Chang *et al.*^32^ has shown that EA enhanced insulin sensitivity by rising glucose tolerance and lowering FBS levels in diabetic rats. In consist, our findings support our hypothesis in that Metformin and acupuncture combined therapy has a significant influence on insulin sensitivity as a kind of insulin sensitizer, and is more effective than Metformin monotherapy.

On another note that since obesity-induced IR is the predominant factor underlying both metabolic syndrome and the rising tide of T2DM,^3^ and that weight loss among overweight/obese people may prevent/control IR and T2DM, we have suggested that acupuncture can improve insulin sensitivity and T2DM by reducing body weight.

Regarding visceral obesity, there is a correlation between increased FFAs and IR. In fact, FFA is one of the key factors that influence insulin activity, and elevated FFA levels are predictive of the progression from impaired glucose tolerance to clinical diabetes.^33^ Insulin is a potent inhibitor of lipolysis and suppresses the release of FFAs from the adipocyte by inhibiting hormone-sensitive lipase.^34^ High levels of circulating FFAs, which can be found in obese individuals, are thought to cause IR in the fibers of skeletal muscles. In addition, increased ceramides biosynthesis, induced by TNF-α and saturated FFAs, seem to impair insulin action on glucose uptake and glycogen synthesis.^1^ Thus, the discovery of new targets that regulate FFAs in adipocytes might ultimately lead to therapeutic modalities, such as acupuncture, that can aid in weight loss and prevent IR and T2DM. In our study, we were interested in the specialized influence of the lipid profiles on insensitivity, when obese/overweight T2DM patients are treated with acupuncture-combined therapy. Concerning the results of the lipid profiles, positive changes in both case and control groups were observed, and the levels of HDLc increased, while the levels of other parameters including TG, LDLc, FFAs and ceramides decreased. The changes were significantly different only in the case group, indicating that while Metformin had effects on the lipid profiles, the acupuncture and Metformin combined therapy leads to a further improvement in lipid profile levels. Moreover, EA may be able to reduce dyslipidemia, which is often found in T2DM individuals. In several studies, a similar pattern of changes following acupuncture in TG, LDLc and HDLc has been reported, which is consistent with our study.^35^ However, two of these studies did not find any changes in HDLc,^30, 31^ which may be due to the application of different acupoints. Significant changes seen in the lipid profiles in our study between case and control groups may confirm a possible systemic effect of EA in reversing dyslipidemia, especially by the modulation of FFA levels, which can in turn influence insulin secretion and IR in T2DM. Our result are similar to another study, in which, they have assessed, that EA restores divergent adipose-tissue gene expression associated with IR, obesity and inflammation.^17^ As a result, we conclude that EA may prevent IR as a kind of insulin sensitizer, by also improving lipid metabolism mechanism. In addition, EA may be able to treat obesity by altering lipid profiles.

Adipokines such as leptin, adiponectin, serotonin, resistin and GLP-1, which are secreted by adipocytes or adipose tissues, can result in low-grade inflammation and have important roles in central obesity, IR and T2DM. In fact, they can regulate body weight, appetite and energy expenditure and modulate insulin sensitivity.^36^ Leptin is involved in the regulation of energy homeostasis, and a relationship exists between leptin and the low-grade inflammatory state of obesity.^15^ Leptin resistance occurs during the early stages of obesity and greatly influences the metabolism of muscle fatty acids and insulin sensitivity.^16^ Adiponectin enhances insulin sensitivity and lipid oxidation, and has vascular protection effects, whereas resistin, whose level is increased in obesity, may induce IR. In addition, serotonin-induced secretion of β-endorphin from the adrenal gland results in decreased plasma glucose levels.^5^ Chang *et al.*^37^ reported EA's effect on increasing plasma β-endorphin and insulin levels, which could interfere with the release of serotonin and induce hypoglycemia. To the best of our knowledge, there has not been any RCT to evaluate efficacy of EA on resistin, and GLP-1 for weight loss and IR, and there are only a few RCTs that study the effect of EA on leptin and adiponectin. Luo and Li^38^ have shown a significant decrease in leptin and an increase in adiponectin in the EA group in comparison with the sham group. According to our results, all measured adipokines were improved in both two groups, but changes were significant only in the case group, suggesting that the efficacy of EA on adipokines may be due to the simultaneous weight loss and modulation of IR. In conclusion, EA and Metformin combined therapy is more effective in changing adipokine levels in comparison with Metformin monotherapy, and EA is able to improve insulin sensitivity also through improving adipokines mechanism.

In summary, our findings suggest that Metformin and acupuncture combined therapy is more effective than Metformin monotherapy on weight loss and improve insulin sensitivity among overweight/obese T2DM patients. EA may be able to treat obesity through other different mechanisms as well, such as the suppression of inflammation and the improvement of lipid metabolism. In addition, EA may improve insulin sensitivity and T2DM through different mechanisms, such as weight loss, its anti-inflammatory effects, and the improvement of lipid metabolism and adipokines. Our study suggests that EA may be considered as a new insulin sensitizer, which may control the epidemics of obesity and T2DM in parallel, to provide longer, healthier lives to people diagnosed with T2DM and to those who are at risk for developing this disease in the future. For our next challenge, we need to evaluate the possible benefits of EA combined therapy in preventing IR in T2DM patients for a longer-term study with a larger sample size.^39, 40, 41, 42, 43, 44, 45, 46, 47, 48^

## Figures and Tables

**Table 1:** Comparison of clinical and biochemical characteristics of participants

*Variable index*	*Case group*			*Control group*			P*-value difference*
	*First sample*	*Second sample*	*Last sample*	*First sample*	*Second sample*	*Last sample*	
Body weight (kg)	82.6±6	-	78.4±6a,^b^	83.5±5	-	82.0±5	*P*<0.001
BMI (kg m^−^^2^)	27.6±2.5	-	26.2±2.4a,^b^	28.2±1.6	-	27.7±1.6	*P*<0.001
FBS (mmol l^−1^)	6.65±0.6	6.44±0.5	6.12±0.5a,^b^	6.67±0.5	6.55±0.5	6.54±0.5	*P*<0.001
FINS (μIU ml^−1^)	14.47±0.8	12.92±0.7	9.91±0.7a,^b^	14.45±0.7	13.75±0.7	13.6±0.8	*P*<0.001
HOMA	4.25±0.2	3.68±0.1	2.67±0.1a,^b^	4.36±0.6	4.06±0.5	4.01±0.5	*P*<0.001
FFAs (mmol l^−1^)	0.72±0.04	0.61±0.03	0.51±0.03a,^b^	0.72±0.04	0.70±0	0.63±0.04	*P*<0.001
TG (mmol l^−1^)	2.59±0.6	2.44±0.6	2.24±0.5a,^b^	2.75±0.7	2.69±0.7	2.65±0.7	*P*<0.001
LDLc (mmol l^−1^)	4.05±0.5	3.88±0.5	3.68±0.5a,^b^	4.08±0.5	4.0±0.5	3.95±5	*P*<0.001
HDLc (mmol l^−1^)	1.08±0.1	1.20±0.1	1.29±0.1a,^b^	1.16±0.1	1.21±0.1	1.25±0.1	*P*<0.001
Ceramides (g dl^−1^)	8.06±0.2	7.24±0.3	6.06±0.3a,^b^	7.92±0.2	7.45±0.2	7.18±0.2	*P*<0.001
TNF-α (pg ml^−1^)	1.44±0.02	1.42±0.04	1.41±0.05a,^b^	1.44±0.02	1.44±0.02	1.43±0.02	*P*=0.04
IL-6 (pg dl^−1^)	1.44±0.03	1.30±0.04	1.09±0.05a,^b^	1.43±0.02	1.41±0.02	1.37±0.04	*P*<0.001
CRP (mg dl^−1^)	0.60±0.03	0.59±0.03	0.57±0.03a,^b^	0.59±0.03	0.58±0.03	0.57±0.03	*P*=0.082
Leptin (ng ml^−1^)	13.32±0.2	11.58±0.4	10.84±0.4a,^b^	13.35±0.2	12.67±0.4	12.60±0.4	*P*<0.001
Adiponectin (μg ml^−1^)	7.47±0.2	8.26±0.2	8.73±0.2a,^b^	7.48±0.3	7.58±0.3	7.73±0.3	*P*<0.001
GLP-1 (mmol l^−1^)	3.77±0.2	3.49±0.2	3.21±0.1a,^b^	3.75±0.2	3.50±0.2	3.43±0.2	P<0.001
Resistin (ng ml^−1^)	7.43±0.2	6.34±0.3	5.24±0.3a,^b^	7.36±0.2	6.76±0.2	6.54±0.1	*P*<0.001
Serotonin (ng ml^−1^)	130.4±3	144.9±2	170.5±3a,^b^	128.0±2	135.1±2	147.0±3	*P*<0.001

Abbreviations: BMI, body mass index; CRP, C-reactive protein; FBS, fasting blood sugar; FFAs, free fatty acids; FINS, fasting insulin; GLP-1, glucose-like peptide-1; HDL-C, high-density lipoprotein cholesterol; IL-6, interleukin-6; LDL-C, low-density lipoprotein cholesterol; TG, triglyceride; TNF-α, tumor necrosis factor. Values are exprsessed as mean±s.e.m.

a Means significant changes in comparison with first sample.

b Means significant changes in comparison with the other group.
